# Measuring health-related quality of life in cardiovascular disease using a novel patient-centred and disease-specific patient-reported outcome measure^☆^

**DOI:** 10.1016/j.ijcrp.2024.200357

**Published:** 2024-12-11

**Authors:** Tajinder K. Singh, Daan Ties, Hilde E. Groot, Paul F.M. Krabbe, Pim van der Harst

**Affiliations:** a Department of Cardiology, University of Groningen, University Medical Center Groningen, Groningen, the Netherlands; b Department of Epidemiology, University of Groningen, University Medical Center Groningen, Groningen, the Netherlands; c Department of Cardiology, Division of Heart & Lungs, Utrecht University, Utrecht University Medical Center, Utrecht, the Netherlands; d Château Santé, Zeist, the Netherlands

**Keywords:** Health-related quality of life, Health status, Patient-reported outcome measure, Preference-based, Cardiovascular disease, Quality of care

## Abstract

**Background:**

Assessment of health-related quality of life (HRQoL) in patients with cardiovascular disease (CVD) is impaired by limitations of current patient-reported outcome measures (PROMs). We developed the first cardiovascular disease (CVD) specific electronic PROM for which health items were derived by a fully patient-centered method. This paper reports on the measurement of HRQoL in CVD patients by a novel developed electronic patient-centred PROM based on a preference-based measurement model.

**Methods and results:**

In an earlier patient-based study nine health items were selected as most important to CVD patients. These items were assessed in the novel preference-based PROM of this study. CVD patients registered with a Dutch patient organization were asked to rate their health state. We compared HRQoL between subgroups of age, gender and CVD. A total of 554 patients participated in this study. The patient reported health items “worry”, “self-reliance” and “sexuality” had the highest impact on HRQoL of CVD patients. Median HRQoL was better for men compared to woman (−17.04, IQR: 31.47 to −3.91 vs. −25.22; IQR: 42.06 to −9.53, p = 0.003). Best and worst HRQoL were observed in patients with an unknown or other CVD disease (−15.61, IQR: 28.52 to −3.91) followed by individuals with coronary artery disease (−16.99, IQR: 38.08 - 0.00) and heart failure (−24.27, IQR: 42.64 to −12.98).

**Conclusions:**

This novel patient-centred, preference-based, CVD-specific PROM accurately measures HRQoL by taking individual health preferences into account and tackling limitations of current PROMs. This PROM is therefore promising to evaluate interventions and optimize personalized therapies.

## Highlights

• By addressing limitations of current PROMs our novel PROM allows for a more accurate HRQoL quantification of individuals suffering from cardiovascular disease by involvement of patients in the PROM's development and tailored weighting of health item levels.
• The patient reported health items “worry”, “self-reliance” and “sexuality” had the highest impact on HRQoL of CVD patients.
• The best HRQoL was observed in males and patients with an unknown or other CVD disease followed by individuals with coronary artery disease. Worst HRQoL was observed in woman and patients with heart failure.

## Graphical abstract

**Figure:**
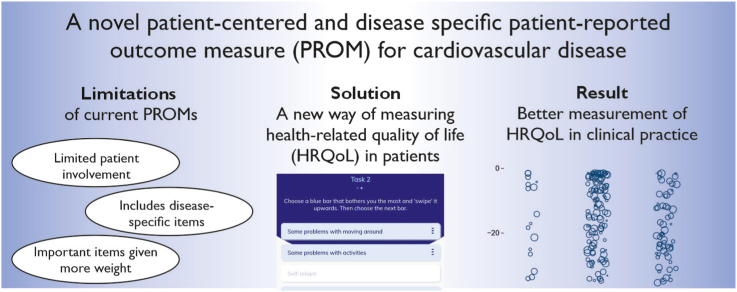

## 1 Introduction

Cardiovascular disease (CVD) mortality has declined considerably over the past 30 years due to reduction of risk factors and advances in medical treatment [1]. However, the burden of CVD remains high as patients face the impact of CVD for a longer time. Improvement of health status as perceived by patients, often referred to as ‘health-related quality of life’ (HRQoL), has become important in management of CVD patients, as ‘quality of life’ is valued more important than length of life [[2], [3], [4]]. HRQoL is a multidimensional concept which reflects an individual's own perceived well-being on different domains of health, such as physical, mental, emotional and social domains [5].

Patient-reported outcome measures (PROMs) are used to measure HRQoL, but many existing PROMs applied to CVD patients have significant limitations. First, commonly used PROMs often involve limited patient input during their development (content) and tend to reflect the perspectives of medical professionals [6,7]. When patients are not actively involved throughout the entire development process of PROMs, including selection of health items, the content will not be clinically relevant or reflect real experiences and concerns of CVD patients. Second, most PROMs are generic tools that focus on overall health rather than the specific effects or symptoms of particular diseases [[8], [9], [10]]. Third, health items and their levels (e.g., no pain, some pain, moderate pain, severe pain) are equally weighted in calculating HRQoL scores in conventional PROMs, neglecting individual patient preferences. Impact of various health aspects can differ across patients with CVD. As a result, the impact of potentially effective interventions may go unnoticed. To overcome limitations of current instruments that measure HRQoL, we developed a novel CVD-specific, patient-centred and preference-based PROM [9]. The present study aims to demonstrate the PROM, generate weights for each level of health items for calculation of HRQoL and report HRQoL measures of CVD patients obtained by the novel instrument.

## 2 Methods

### 2.1 Study design and participants

Individuals registered with Harteraad, the largest Dutch patient organization for people with CVD, were recruited for this study [11]. Participants were asked to complete two tasks to assess HRQoL. First, patients had to rate their health on nine health items. Second, patients were asked to make multiple selections which of the nine health descriptions disturbed them the most. At last, demographic questions were asked regarding age, gender and type of cardiovascular disease which were self-reported by patients. The Medical Ethical Committee of the University Medical Center Groningen approved the protocol and waived the need for written informed consent because this research did not fall under the scope of the Medical Research Involving Human Subjects Act (METc 2019/538). The execution of this study complied with the Declaration of Helsinki.

### 2.2 Novel PROM

The novel PROM was administered through an online software application, the HealthSnApp (www.healthsnapp.info). A previous study was conducted to generate content for the PROM by first identifying existing health items through a scoping literature review, interviews with CVD patients and having a first expert group meeting. Second, patients were asked in an online survey to indicate the health items they considered most important from the constructed graphical overview of health items [9]. Based on these results and a second-round expert group meeting, the nine items deemed most important for health by CVD patients were incorporated in the electronic PROM: “mobility”, “activities”, “self-reliance”, “fatigue”, “shortness of breath”, “chest pain”, “palpitations”, “anxiety/worrying”, and “sexual limitations”.

### 2.3 Measurement model

Our work is based on a novel preference-based measurement model, named multi-attribute preference response model (MAPR) which uses an indirect approach for measurement [12,13]. Ordinal response data (ranks) gathered from specific preference tasks are aggregated to estimate coefficients within a mathematical measurement model. This model consists of a latent (hidden) variable (the metric scale) and a set of manifest (observable) variables (i.e., the items of the PROM). The MAPR model, therefore, relies on a probabilistic, group-based measurement approach, drawing from the aggregated responses of patients. This methodology has a long history, originating with Louis Thurstone's 1927 model, and has been further developed by various researchers. For these models to function properly, respondents must engage in assessments (processing information before making a judgment) and judgments (choosing between options) in a specific way, generating the necessary data for analysis. Assessments typically involve comparing at least two objects, such as health states or health items, to determine preferences. To collect this data, we used the Drop-Down method (Task 2 below), which is designed to produce preference data that fits within this probabilistic measurement framework.

### 2.4 HealthSnApp

**Fig. 1:**
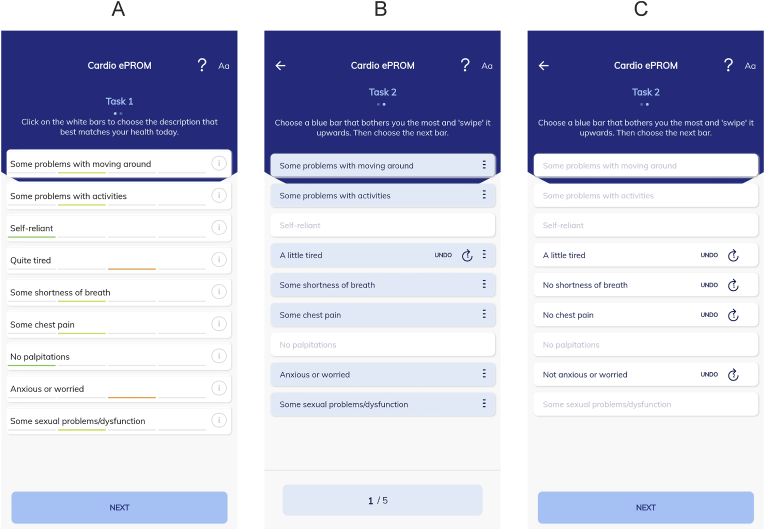
Screenshots of the novel cardiovascular-disease-specific, patient-centred and preference-based patient-reported outcome measure. The ePROM consists of nine items considered most important by cardiovascular disease patients. (A) In the first task respondents were presented with a screen with nine boxes corresponding to nine health items considered important by cardiovascular disease patients. They were asked to assess their current health state by rotating the boxes till the best fitting descriptions were obtained on all health items. For example, when the box labelled “anxiety or worry” was selected, the description changed to the following response options: “not worried or anxious”, “slightly worried or anxious”, “worried or anxious” and “highly worried or anxious”. (B) In the second task respondents were presented with the health description defined in task 1. They were asked to select a maximum of 5 times which of the nine health item-levels descriptions disturbed them the most. Blue coloured boxes displayed that there were problems regarding the health item. For instance, in the screenshot “fatigue” was swiped, which moved the level of the item one level better from “quite tired” to “a little tired”. (C) Screen after 5 dropdowns of health items with a particular level.

*Task 1: defining individual (reference) health state* In the first task patients were presented with a screen showing the nine health items. Each item consisted of four levels of severity, ranked from no problems to very severe. For example, the health item fatigue consisted of the following levels: not tired, a little tired, quite tired and very tired. By clicking once on a box corresponding to a health item, the box rotated to display the first severity of a health item (e.g., not tired). Clicking once again on the box would display the second severity of a health item (e.g., a little tired) and so forth. Patients were asked to provide their current health state by rotating the boxes till the best fitting descriptions were obtained on all health items (Fig. 1a). In this manner the HealthSnApp expressed the patients’ health state as nine digits (e.g., 221322132). Each individual digit represents the level of severity of a health item. This first task defines a reference health state for the next task.

*Task 2: Drop-Down task* In the second preference-based task patients were first presented with their own (reference) health state. White boxes displayed that patients did not face any issues regarding the health item presented and blue boxes displayed patients experienced some level of problem regarding the health item (Fig. 1b). Patients were asked fives time to select a blue box from which they suffered the most of or preferred the least and had to swipe this box upwards. After swiping the blue box, the description of the box changed to one level less severe of the health item. In this way a new improved health state was constructed. For example, a patient with a reference health state of 221322132 could swipe the blue box with the description quite tired to the description a little tired, changing the health state to 221222132. Next, a patient could change the health state to 221222122 by swiping the blue box with the description anxious or worried to slightly anxious or worried. Items at level 3 or higher could be dropped down more than once. By performing this task rankings of health states are generated that can be used to estimate weights for each level of the nine items. Defining weights for each level of an item enables to yield a single score for overall HRQoL by adding up coefficients of a certain health state. Patients who reported having a perfect health state (111111111) in the first task did not take part in the second task.

### 2.5 Statistical analysis

Coefficients (weights) for the levels of each health item were estimated using a rank-ordered logit model (Stata, cmrologit). The first level of each health item (no problems) was taken as the reference category. Negative coefficients implied that a particular level was worse than the reference, which was the first level of each health item. Moreover, the less preferable a level was considered, the higher its coefficient was in a negative direction. Results were reported as means [± standard deviation (SD)] for continuous normally distributed variables, median [interquartile range (IQR)] for non-normally distributed variables, and frequencies (%) for categorical variables, and compared between subgroups using independent *t*-tests, Mann–Whitney U-tests, or χ^2^ tests. Patients were divided into three age groups: <60 years, 60–70 years and >70 years. Number of responses on the levels for each of the health items were calculated and compared between subgroups of CVD using χ^2^ tests. HRQoL were compared between CVD-subgroups, gender and age using Mann-Whitney *U* test. Data was analysed and visualized using STATA (version 17.0, StataCorp LCC, College Station, USA), CorelDraw and GIMP version 2.10.

## 3 Results

**Completion and patient characteristics** Invitations to participate in this study were sent to 2600 patients. A total of 554 participants responded and were subsequently recruited. The first task was completed by all 554 respondents. As 80 respondents defined their reference health state as perfect (111111111) in the first task, 474 respondents went on to the second task. The survey was completed by 423 respondents, while 118 respondents did not fill it in (Figure S1). The mean age of respondents was 66.1 ± 10.8 years, with 36.2 % being female. The most common diagnosis among respondents was categorized as an unknown or other CVD diagnosis, followed by HF and CAD (Table 1).

**Table 1:** Baseline characteristics of study population (n=436^a^).

Characteristics	Total population n = 436^a^	Suboptimal health state^b^ n = 357	Optimal health state^c^ n = 79	P-value
Age (years), mean ± SD	66.1 (10.8)	65.6 (11.1)	68.6 (9.2)	0.015^d^
Age groups, n (%)				0.040^d^
< 60	107 (25.2)	95 (27.3)	12 (15.6)	
60-70	155 (36.5)	128 (36.8)	27 (35.1)	
> 70	163 (38.3)	125 (35.9)	38 (49.3)	
Gender, n (%)				0.026^d^
Male	278 (63.8)	219 (61.3)	59 (74.7)	
Female	158 (36.2)	138 (38.7)	20 (25.3)	
Cardiovascular disease, n (%)				0.047^d^
Coronary artery disease	82 (18.8)	61 (17.1)	21 (26.6)	
Heart failure	96 (22.0)	88 (24.7)	8 (10.1)	
Congenital heart disease	21 (4.8)	18 (5.0)	3 (3.8)	
Cardiac arrhythmia	67 (15.4)	55 (15.4)	12 (15.2)	
Heart valve disease	21 (4.8)	15 (4.2)	6 (7.6)	
Others/unknown	149 (34.2)	120 (33.6)	29 (36.7)	

Data are expressed as mean ± standard deviation (SD) or as number (%).

a Of 554 respondents, 436 participants filled in one or more questions regarding personal information. Of the 436 participants, 11 participants did not fill in the question regarding age.

b Participants completed task 1 and 2.

c Participants only completed task 1 as their health state score was optimal (111111111).

d P-value below the statistical significance threshold of 0.05.

### 3.1 Frequency of defined health statuses by respondents

The most frequently reported health item by all respondents and by every subgroup of CVD was “fatigue”. A total of 396 (70%) respondents experienced fatigue. The least frequently reported item by respondents was “self-reliance”. Most individuals (78%) were self-reliant. There were significant differences observed in frequencies of reported health items among subgroups of CVD. Compared to other subgroups, individuals with HF experienced more often shortness of breath (66%, p = 0.004) and individuals with cardiac arrhythmia experienced more often palpitations (43%, p < 0.001) (Supplementary Table 1).

### 3.2 Coefficients

Responses from 474 participants were included in the regression analysis to estimate the coefficients for each level of health items based on the drop-down task. Coefficients were negative and statistically significant (p < 0.001) for all levels of the nine health items. Negative coefficients were expected in the regression analysis as each level of an item was worse than the reference level, which in our study was the first level of an item. Also, coefficients followed a logical order. Coefficients became more negative as the level of severity of an item increased (Table 2). The response level “highly worried” (−18.25) within the item “worry” had the highest impact on HRQoL of CVD patients, followed by “fully dependent” (−16.60) within the item “self-reliance” and “severe sexual limitations” (−16.38) within the item “sexuality” (Table 2). The response item “some shortness of breath” had the lowest coefficient (−3.62).

**Table 2:** Coefficients for the levels of the nine health items of the cardiovascular patient-reported outcome measure.

**Level of health item**	Coefficient	SE	Significance
**Mobility (2)**	−3.846	0.35	<0.001
**Mobility (3)**	−9.120	0.54	<0.001
**Mobility (4)**	−14.056	0.96	<0.001
**Activities (2)**	−3.784	0.32	<0.001
**Activities (3)**	−8.932	0.51	<0.001
**Activities (4)**	−13.759	0.84	<0.001
**Self-reliance (2)**	−4.230	0.47	<0.001
**Self-reliance (3)**	−9.346	0.74	<0.001
**Self-reliance (4)**	−16.599	1.83	<0.001
**Fatigue (2)**	−3.907	0.30	<0.001
**Fatigue (3)**	−9.191	0.50	<0.001
**Fatigue (4)**	−15.898	0.79	<0.001
**Shortness of breath (2)**	−3.615	0.30	<0.001
**Shortness of breath (3)**	−8.234	0.51	0.000
**Shortness of breath (4)**	−14.758	0.84	0.000
**Chest pain (2)**	−3.843	0.39	<0.001
**Chest pain (3)**	−9.534	0.64	<0.001
**Chest pain (4)**	−16.087	1.19	<0.001
**Palpitations (2)**	−3.636	0.38	<0.001
**Palpitations (3)**	−9.155	0.65	<0.001
**Palpitations (4)**	−15.693	1.61	<0.001
**Anxiety or worry (2)**	−4.232	0.29	<0.001
**Anxiety or worry (3)**	−9.861	0.61	<0.001
**Anxiety or worry (4)**	−18.250	1.59	<0.001
**Sexuality (2)**	−4.227	0.38	<0.001
**Sexuality (3)**	−9.775	0.69	<0.001
**Sexuality (4)**	−16.380	1.32	<0.001

### 3.3 Health state values

**Fig. 2:**
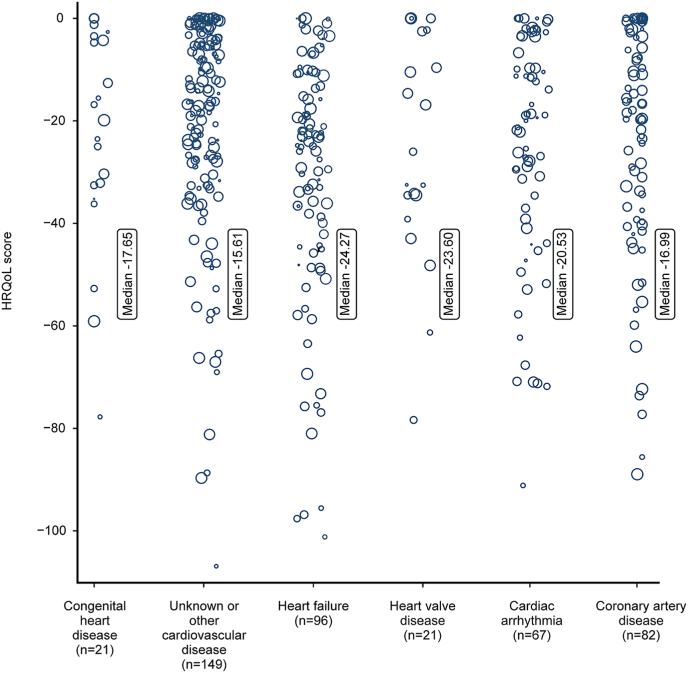
Distribution of health-related quality of life measures (0 = full health, −100 = worse health) obtained for subtypes of cardiovascular disease patients with the patient-reported outcome measure (The size of the circles represents the frequency weight of health-related quality of life measures).

**Fig. 3:**
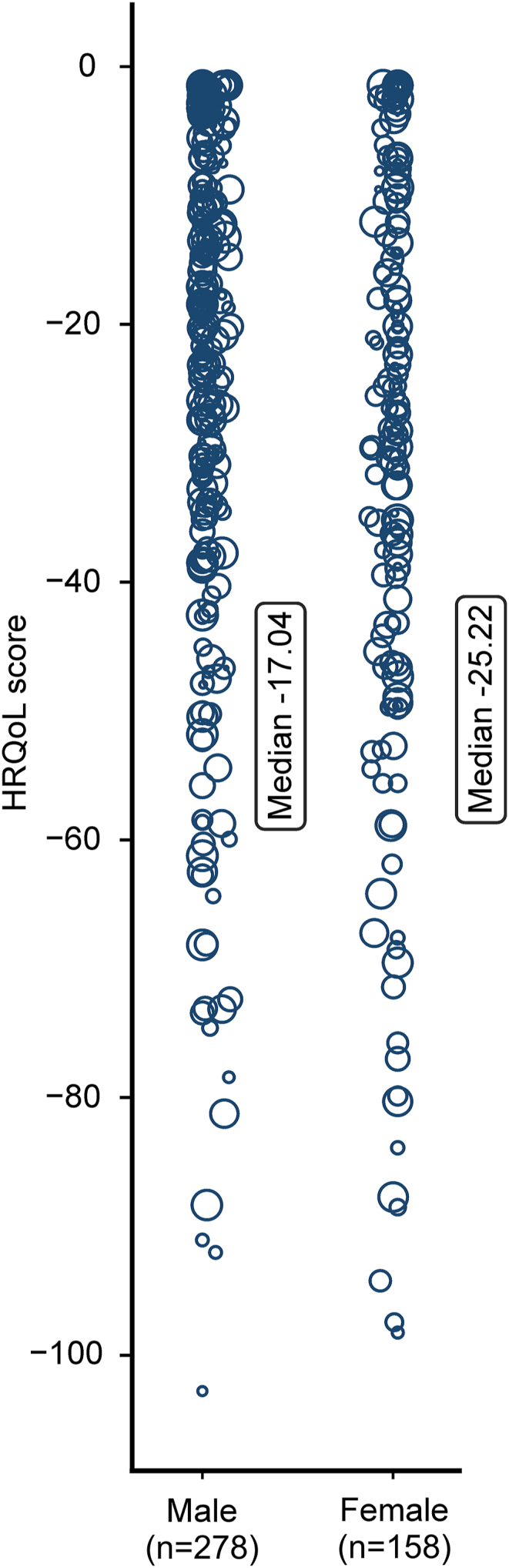
Distribution of health-related quality of life measures obtained for male and female respondents suffering from cardiovascular disease with the patient-reported outcome measure (The size of the circles represents the frequency weight of health-related quality of life measures).

Health-state values among respondents ranged from −3.61 to −106.90. The best health state (111111111) was observed among 80 (14.4 %) respondents (Fig. 2). Median HRQoL was better for men compared to women (−17.04, IQR: 31.47 to −3.91 vs. −25.22; IQR: 42.06 to −9.53, p = 0.003) (Fig. 3). Median HRQoL of individuals with age <60 years was worse compared to 60–70 years (−21.96, IQR: 39.78 to −11.31 vs. −17.43, IQR: 31.94 to −4.23, p = 0.047). No differences in median HRQoL were observed between individuals with age <60 years compared to individuals >70 years (−21.96, IQR: 39.78 to −11.31 vs.-17.65, IQR: 34.78 to −3.64, p = 0.096). Median HRQoL differed between subgroups of CVD diagnosis. Median HRQoL was worse for individuals with HF compared to individuals with CAD (−24.27, IQR: 42.64 to −12.98 vs. −16.99, IQR: 38.08 - 0.00, p = 0.010) and compared to individuals with an unknown or other CVD diagnosis (−24.27, IQR: 42.64 to −12.98 vs. −15.61, IQR: 28.52 to −3.91, p < 0.001).

## 4 Discussion

This study demonstrates a novel patient-centered, preference-based, CVD-specific PROM and reports on measurement of HRQoL in CVD patients. For the first time, patients were involved in every part of the development and individual health preferences were taken into account to accurately measure HRQoL. This novel PROM measured differences in HRQoL between subgroups of patients. Women suffering from CVD experienced worse HRQoL compared to men, HF patients experienced worse HRQoL compared to other CVD patients and CVD patients with age <60 years experienced worse HRQoL compared to those aged 60-70 years.

Recently, comparable preference-based PROMs based on the same measurement framework were used to measure HRQoL [[14], [15], [16]]. These previous studies showed that a preference-based method reliably quantifies HRQoL. Measurement of HRQoL by current PROMs neglect individual health preferences as each health domain and level of severity of a health item is equally weighted, assuming that each health item has equal impact on HRQoL. However, we illustrated by applying a preference-based model that CVD patients find the impact of the item “worry” on HRQoL more important compared to the other eight items. Also, a change in level from worried to highly worried has a greater impact on HRQoL compared to a change of level from not worried to slightly worried. Therefore, by addressing limitations of current PROMs by involvement of patients in the PROM's development and tailored weighting of health item levels, this novel PROM allows for a more accurate HRQoL quantification to guide treatment decisions and evaluate effectiveness of therapies. In our study, we observed that the top three health items “worry”, “self-reliance” and “sexuality” have the highest impact on HRQoL of CVD patients. This means that a change in these items to one level better has the greatest impact on improving overall HRQoL. Although the item “fatigue” was the most common reported problem by participants in the first task, it was not considered to have the highest impact on HRQoL. Previous studies have shown that fatigue is a frequent complaint with CVD [17,18]. Furthermore, we observed that HRQoL measures between subgroups of age, gender and subtypes of CVD differed. We observed that women have significantly worse HRQoL compared to men suffering from CVD, which is in line with previous studies [19,20]. Also, previous research supports that individuals with HF report worse HRQoL compared to CAD and with an unknown or other CVD [21,22]. We observed that individuals with HF reported more often problems on mobility, activities, fatigue, shortness of breath, worry, palpitations and sexuality compared to other CVD patients. At last, we found that younger CVD patients report worse HRQoL compared to the oldest age group (>70 years of age), which is consistent with other studies [23,24] This difference may be explained by the fact that CVD has a bigger impact on younger, and more active, patients than retired older patients. Other previous research reports a negative relationship between HRQoL and increasing age [25,26]. This inconsistency among studies may be accounted to the number of older patients included with a higher degree of comorbidities.

### 4.1 Future perspectives

Our study illustrates that PROMs developed with patient input which takes individual health preferences into account are of importance to accurately measure HRQoL. This novel patient-centered preference-based PROM developed specific for CVD patients has great potential to reliably measure HRQoL to evaluate the effects of interventions and identify from which interventions CVD patients might benefit from. However, we should first conduct further prospective validation by comparing our novel PROM with existing established tools before recommending implantation in routine clinical care.

### 4.2 Limitations

Some limitations of our study should be acknowledged. First, there was a greater proportion of men in our study. However, this does reflect the natural distribution of sexes in CVD patients [27,28]. Second, we applied the PROM in a single patient organization, potentially limiting the generalizability of the findings. However, since Harteraad is the largest Dutch patient organization for individuals with cardiovascular disease, participants were recruited from alle subtypes of cardiovascular conditions, making the sample representative of the overall population. Third, the results of the HRQoL comparisons between subgroups should be interpreted with caution, as no power analysis was conducted. Additionally, participants could not specify the exact subtype of cardiovascular disease in the survey part of the HealthSnApp if they selected the ‘other/unknow’ option in the multiple-choice question. Consequently, we do not know which specific diseases were represented by this group. Nevertheless, this does not impact the study's conclusion, as the primary aim was to demonstrate our PROM, generate weights for each health item level to calculate overall HRQoL, and present initial results of HRQoL measurement in CVD patients. Fourth, since our PROM was delivered via online software, non-response may have been due to participants feeling technically unqualified to complete the task.

## 5 Conclusion

Our novel patient-centered, preference-based, CVD-specific PROM is able to yield a single measure that accurately reflects overall HRQoL of CVD patients by taking individual health preferences into account and tackling limitations of current PROMs. Therefore, it is promising to identify therapies for CVD that truly make an impact on CVD patients and can be tailored to their preferences.

## CRediT authorship contribution statement

**Tajinder K. Singh:** Writing – review & editing, Writing – original draft, Visualization, Validation, Project administration, Methodology, Investigation, Funding acquisition, Formal analysis, Data curation. **Daan Ties:** Writing – review & editing, Validation, Project administration, Methodology, Investigation, Formal analysis, Data curation, Conceptualization. **Hilde E. Groot:** Writing – review & editing, Validation, Project administration, Methodology, Investigation, Conceptualization. **Paul F.M. Krabbe:** Writing – review & editing, Visualization, Supervision, Software, Resources, Project administration, Methodology, Funding acquisition, Conceptualization. **Pim van der Harst:** Writing – review & editing, Validation, Supervision, Resources, Project administration, Methodology, Funding acquisition, Conceptualization.

## Data availability

Data requests should be submitted to the principal investigator (P.vdH.) for consideration. We aim to share data to the maximum extent, but within specific boundaries relating to ethical approval, contractual and legal obligations of this study, and publication timelines. All proposals will be reviewed for their scientific merit by the trial management group. Only data relevant to the purpose of the data request will be provided.

## Funding

This work was supported by the Dutch Heart Foundation (CVON2015-17)

## Declaration of conflicting interests

Paul F.M. Krabbe is the CEO of Château Santé. As an extension of the measurement model presented in this paper, additional measurement models, tools and instruments are developed by Paul F.M. Krabbe as part of academic/commercial activities. The other authors declare no conflict of interest relevant to this article.
