# Supplementary material for: Measuring health-related quality of life in cardiovascular disease using a novel patient-centred and disease-specific patient-reported outcome measure
Source: Int J Cardiol Cardiovasc Risk Prev. 2024 Dec 11;24:200357. doi: 10.1016/j.ijcrp.2024.200357 (PMC11720887; doi:10.1016/j.ijcrp.2024.200357)
Supplement: Multimedia component 1 [file mmc1.docx]

**Supplementary Fig. 1** Flow chart of study population

Did not fill in survey
(n=118)

T

**Excluded**
No response (n=2,046)

Total patients included (n=554)

T

**Harteraad**
Total patients recruited (n=2,600)

T

Filled in one or more questions of survey
(n=436)

T

Completed task 1
(n=554)

T

Completed task 2
(n=474)

T
