# Supplementary material for: Measuring health-related quality of life in cardiovascular disease using a novel patient-centred and disease-specific patient-reported outcome measure
Source: Int J Cardiol Cardiovasc Risk Prev. 2024 Dec 11;24:200357. doi: 10.1016/j.ijcrp.2024.200357 (PMC11720887; doi:10.1016/j.ijcrp.2024.200357)
Supplement: Multimedia component 2 [file mmc2.docx]

**Supplementary table 1.** Frequency distribution of respondent’s health state description

| Health items and levels | All (n=554) | CAD (n=82) | HF (n=96) | CHD (n=21) | | Cardiac arrhythmia (n=67) | Heart valve disease (n=21) | Others or unknown (n=149) | |
| --- | --- | --- | --- | --- | --- | --- | --- | --- | --- |
| Mobility N (%) p = 0.320 |  |  |  |  | |  |  | |  |
| No problems with moving around | 292 (53) | 51 (62) | 42 (44) | 12 (57) | | 39 (58) | 10 (47) | | 86 (58) |
| Some problems with moving around | 151 (27) | 20 (25) | 28 (29) | 6 (29) | 16 (24) | | 9 (43) | | 39 (26) |
| Moderate problems with moving around | 75 (14) | 6 (7) | 21 (22) | 2 (9) | 9 (13) | | 1 (5) | | 15 (10) |
| Severe problems with moving around | 36 (6) | 5 (6) | 5 (5) | 1 (5) | 3 (5) | | 1 (5) | | 9 (6) |
| Activities N (%) p = 0.185 |  |  |  |  |  | |  | |  |
| No problems with activities | 252 (45) | 42 (51) | 31 (32) | 10 (48) | 32 (48) | | 10 (48) | | 82 (55) |
| Some problems with activities | 169 (31) | 25 (31) | 41 (43) | 9 (43) | 19 (28) | | 6 (28) | | 38 (26) |
| Moderate problems with activities | 101 (18) | 11 (13) | 16 (17) | 2 (9) | 12 (18) | | 4 (19) | | 25 (17) |
| Severe problems with activities | 32 (6) | 4 (5) | 8 (8) | 0 (0) | 4 (6) | | 1 (5) | | 4 (2) |
| Self-reliance N (%) p = 0.960 |  |  |  |  |  | |  | |  |
| Self-reliant | 434 (78) | 69 (84) | 72 (75) | 17 (81) | 56 (84) | | 15 (71) | | 122 (82) |
| Somewhat dependent | 98 (18) | 11 (13) | 20 (21) | 3 (14) | 8 (12) | | 5 (24) | | 21 (14) |
| Largely dependent | 19 (3) | 2 (3) | 3 (3) | 1 (5) | 3 (4) | | 1 (5) | | 5 (3) |
| Fully dependent | 3 (1) | 0 (0) | 1 (1) | 0 (0) | 0 (0) | | 0 (0) | | 1 (1) |

**Supplementary table 1.** *(Continued)*

| Health items and levels | All (n=554) | CAD (n=82) | HF (n=96) | CHD (n=21) | Cardiac arrhythmia (n=67) | Heart valve disease (n=21) | Others or unknown (n=149) |
| --- | --- | --- | --- | --- | --- | --- | --- |
| Fatigue N (%) p = 0.247 |  |  |  |  |  |  |  |
| Not tired | 168 (30) | 33 (40) | 19 (20) | 5 (24) | 23 (34) | 8 (38) | 55 (37) |
| A little tired | 187 (34) | 24 (29) | 38 (40) | 8 (38) | 22 (33) | 6 (29) | 57 (38) |
| Quite tired | 156 (28) | 21 (26) | 31 (32) | 8 (38) | 17 (25) | 5 (24) | 31 (21) |
| Very tired | 43 (8) | 4 (5) | 8 (8) | 0 (0) | 5 (8) | 2 (9) | 6 (4) |
| Shortness of breath N (%)  p = 0.004^a^ |  |  |  |  |  |  |  |
| No shortness of breath | 277 (50) | 47 (58) | 33 (34) | 12 (57) | 36 (54) | 10 (48) | 91 (61) |
| Some shortness of breath | 173 (31) | 19 (23) | 37 (39) | 3 (14) | 18 (27) | 8 (38) | 46 (31) |
| Moderate shortness of breath | 84 (15) | 15 (18) | 19 (20) | 4 (19) | 10 (15) | 2 (9) | 10 (7) |
| Severe shortness of breath | 20 (4) | 1 (1) | 7 (7) | 2 (10) | 3 (4) | 1 (5) | 2 (1) |
| Chest pain N (%) p = 0.111 |  |  |  |  |  |  |  |
| No chest pain | 412 (74) | 50 (61) | 71 (74) | 15 (71) | 54 (81) | 18 (86) | 120 (81) |
| Some chest pain | 114 (21) | 23 (28) | 21 (22) | 4 (19) | 13 (19) | 2 (10) | 22 (15) |
| Moderate chest pain | 21 (4) | 7 (9) | 2 (2) | 2 (10) | 0 (0) | 1 (5) | 5 (34) |
| Severe chest pain | 7 (1) | 2 (2) | 2 (2) | 0 (0) | 0 (0) | 0 (0) | 2 (1) |
| Palpitations N (%) p = <0.001^a^ |  |  |  |  |  |  |  |
| No palpitations | 381 (69) | 62 (76) | 62 (65) | 13 (62) | 38 (57) | 15 (71) | 119 (80) |
| Some palpitations | 132 (24) | 16 (20) | 25 (26) | 7 (33) | 14 (21) | 5 (24) | 28 (19) |
| Moderate palpitations | 31 (5) | 4 (4) | 5 (5) | 1 (5) | 11 (16) | 0 (0) | 2 (1) |
| Severe palpitations | 10 (2) | 0 (0) | 4 (4) | 0 (0) | 4 (6) | 1 (5) | 0 (0) |

**Supplementary table 1.** *(Continued)*

| Health items and levels | All (n=554) | CAD (n=82) | HF (n=96) | CHD (n=21) | Cardiac arrhythmia (n=67) | Heart valve disease (n=21) | Others or unknown (n=149) |
| --- | --- | --- | --- | --- | --- | --- | --- |
| Worry N (%) p = 0.843 |  |  |  |  |  |  |  |
| Not worried | 319 (58) | 50 (61) | 52 (54) | 12 (57) | 36 (54) | 14 (66) | 89 (60) |
| Slightly worried | 179 (32) | 22 (27) | 32 (33) | 9 (43) | 23 (34) | 6 (29) | 45 (30) |
| Worried | 48 (9) | 9 (11) | 9 (10) | 0 (0) | 8 (12) | 1 (5) | 12 (8) |
| Highly worried | 8 (1) | 1 (1) | 3 (3) | 0 (0) | 0 (0) | 0 (0) | 3 (2) |
| Sexuality N (%) p = 0.793 |  |  |  |  |  |  |  |
| No sexual limitations | 312 (56) | 47 (57) | 50 (52) | 13 (62) | 41 (61) | 11 (52) | 93 (62) |
| Some sexual limitations | 104 (19) | 18 (22) | 17 (18) | 5 (24) | 11 (16) | 4 (19) | 29 (20) |
| Moderate sexual limitations | 51 (9) | 7 (9) | 14 (14) | 1 (5) | 5 (8) | 1 (5) | 11 (7) |
| Severe sexual limitations | 87 (16) | 10 (12) | 15 (16) | 2 (9) | 10 (15) | 5 (24) | 16 (11) |

*^a^ P-value below the statistical significance threshold of 0.05.
 Data are expressed as number (%).*
